# Cache Scheme Based on Pre-Fetch Operation in ICN

**DOI:** 10.1371/journal.pone.0158260

**Published:** 2016-06-30

**Authors:** Jie Duan, Xiong Wang, Shizhong Xu, Yuanni Liu, Chuan Xu, Guofeng Zhao

**Affiliations:** 1 Communication and Information Engineering, Chongqing University of Posts and Telecommunications (CQUPT), Chongqing, China; 2 Communication and Information Engineering, University of Electronic Science and Technology of China (UESTC), Chengdu, China; University of South Australia, AUSTRALIA

## Abstract

Many recent researches focus on ICN (Information-Centric Network), in which named content becomes the first citizen instead of end-host. In ICN, Named content can be further divided into many small sized chunks, and chunk-based communication has merits over content-based communication. The universal in-network cache is one of the fundamental infrastructures for ICN. In this work, a chunk-level cache mechanism based on pre-fetch operation is proposed. The main idea is that, routers with cache store should pre-fetch and cache the next chunks which may be accessed in the near future according to received requests and cache policy for reducing the users’ perceived latency. Two pre-fetch driven modes are present to answer when and how to pre-fetch. The LRU (Least Recently Used) is employed for the cache replacement. Simulation results show that the average user perceived latency and hops can be decreased by employed this cache mechanism based on pre-fetch operation. Furthermore, we also demonstrate that the results are influenced by many factors, such as the cache capacity, *Zipf* parameters and pre-fetch window size.

## 1. Introduction

Recently, most of the network applications and services only care about content distribution and retrieval, while the current Internet still relies on the host-to-host communication model. Several researchers devote to Information-Centric Network (ICN) [1–6] in recent years, which is a significant potential approach to resolve such mismatching problem mentioned above. The voice transformed in MANET [7, 8] is one type of the contents. The content objects in ICN are routed by content names, not by host locators (IP address). Named contents become the first citizen in the ICN architecture instead of end-host, which gives the opportunity to identify content objects as they travel from source to destination. In turn, given that named content objects are transferred instead of unidentifiable data containers, i.e., IP packets, these objects can be cached in the network and served to subsequent users with the similar interest.

All ICN architectures proposed share three commonalities [9, 10]. The first one is the publish/subscribe (pub/sub) paradigm. A content provider publishes a content object, while a client subscribes to the content file through describing the corresponding interest. This temporal and spatial decoupling between information generation and interest indication is a desirable feature for future internet. Secondly, the information oriented security model is adopted. Because content objects are signed by the original content provider, the authenticity of content can be easily verified by network elements or consumers. The web application protection techniques in [11–14] can be seen as the typical ICN information oriented security models. The third commonality is the universal in-network cache, which is the main researching concern of this paper. content in ICN can be further divided into many small-sized chunks. Chunk-based communication has merits over content-based communication, for example, user can get a content file from many different sources by the means of chunks, and fine-granularity cache scheme should be designed.

Caching and pre-fetching operation are well-known strategies deployed in application layer for improving the performance of current network. The caching scheme is composed by two key parts: i) Cache decision, which decides on whether an object should or not to be stored; and ii) Replacement policy, which is used to select the replacing objects in cache for the newly replied objects. Compared to the traditional application-layer content distribution system, the network-layer cache platform of ICN has its own prosperities: 1) Universal cache with small capacities. Almost all routers in the network can be configured with small capacity and high-speed memory technology. 2) Interaction relationship between cache configuration and network traffic. The router cache policy or capacity impacts the upward traffic through cache hit rate. Meanwhile, the downward traffic can affect the arriving rate of the cache router to further result in the cache policy or capacity deployment. 3) Provides a unified platform of network layer content caching for various flows. 4) ICN content acquisition mode provides new cache management method. In-network cache policy for ICN has attracted more attentions in last few years [15–23]. Ioannis Psaras et al. [15] determine the caching probability of a content object in each node on the path. Progressive caching [16] sequentially pushes the content to be cached closer to the edge node based on content popularity. The main idea of chunk-level cache scheme WAVE [17] is suggested that routers should dynamically adjust their cache windows for content on the basis of the content popularity, and should recommend the number of chunks to be cached at the downstream node. ABC (Age-Based Cooperation) [18] sets an age for each cached item, which should be evicted from the cache only when the age is expired. Aifang Xu et al. [19] propose use tracking store (TS) to assist caching objects. Jos´e Quevedo et al. [20] use a freshness value to design the cache policy under wireless sensor network. Pending time history is carried in interest for help to compute the better caching set for each router in [21]. Wang, W. et al. [22] exploits the correlation between content popularity and network topology information for ICN caching. ILP is used for programming the caching of the complex dynamic streaming in [23]. The different methods for the above mentioned works were proposed to improve the network performance. However, none of them has involved in pre-fetch operation to further fast the content retrieval.

By the same token, the essence of pre-fetching algorithm relies on its operation, how to pre-fetch, and the pre-fetched objects. Comparing to cache policy, pre-fetch operation is likely a strategy to hide retrieval network latency for the user rather than to reduce it. Pre-fetch operation has been studied in web service last twenty years ago [24–26]. One main drawback in Web pre-fetch system is over-prefetch, that is, when space (cache) on a server is allowed, all the referencing webs should be pre-fetched and cached to be requested. Nevertheless, many pre-fetched objects may not be issued by clients, which waste storage capacity and overlong the total network latency. Path-based pre-fetching and caching are integrated in file system [24]. The result has shown that aggressive pre-fetching is harmful to latency reduction. To offset such defects, most web pre-fetch operations concentrate on the high accuracy prediction model of pre-fetch probability (referencing objects), which include temporal locality, data mining, or more complicated mathematic models. Padmanabhan and Mogul [25] have investigated the tradeoff between network load incremental and performance gain of web pre-fetching based on renewing of dependency graph. Cao et al. [26] have studied an integrated caching and pre-fetch model on a file system from a theoretical viewpoint. The differences between in-network caching for ICN and the web caching have been listed before. Because of the characteristics of universal caching and chunk-based communication in ICN, routers could pre-fetch the following chunks for a given content, avoiding complex predication algorithm in web pre-fetch operation. The shortcoming of over-prefetch should be ignored for the LRU cache replacement in our work. Even though the number of chunks pre-fetched is large enough, the network latency is also shorter than that without pre-fetch operation. The pre-fetching operation for ICN has been studied in [27–29]. The pre-fetching is mentioned in [27] to improve the retransmission and congestion control mechanisms in ICN. But no details or following works are given about the pre-fetch operation. Authors in [28] focus on prefetching content chunks for individual mobile devices, by predicting the next network location the device is moving to, and at what time, and examine how best to partition the storage at the AP. Obviously, the pre-fetching operation here is just activated by APs, and the main contribution is about the predicting of the device’s mobility and the user pattern. [29] harnesses multiple caches in ICN to pre-fetch video stream during peak congestion. Architecture for pre-fetching for video streams is presented, which combines network monitoring with ICN semantics to populate a cache ahead of the need of the video client. Our argument is that all the cache routing should determine the pre-fetch operation, and the focus is how and when to pre-fetch. Moreover, our goal is to reduce the latency experienced by clients by considering cache policy based on pre-fetch operation.

In this paper, the idea of pre-fetching and caching can be perfectly applied into in-network ICN caching together. Cache router can activate the pre-fetch operation, and pre-fetched objects should also be stored on ICN cache routers. Therefore, our main contribution is trying to answer how to design an efficient cache scheme based on pre-fetch operation to reduce latency, and measure the effect of pre-fetch windows for improving performance.

## 2. Preliminary Knowledge

Our cache scheme based on pre-fetch operation can be applied onto any of chunk-level ICN architecture. In order to facilitate the presentation, the CCN (content centric network) [3] is taken as an example. The term “data” and “chunk” can be interchangeable in remain part of this paper, unless specifically stated.

**Fig 1:**
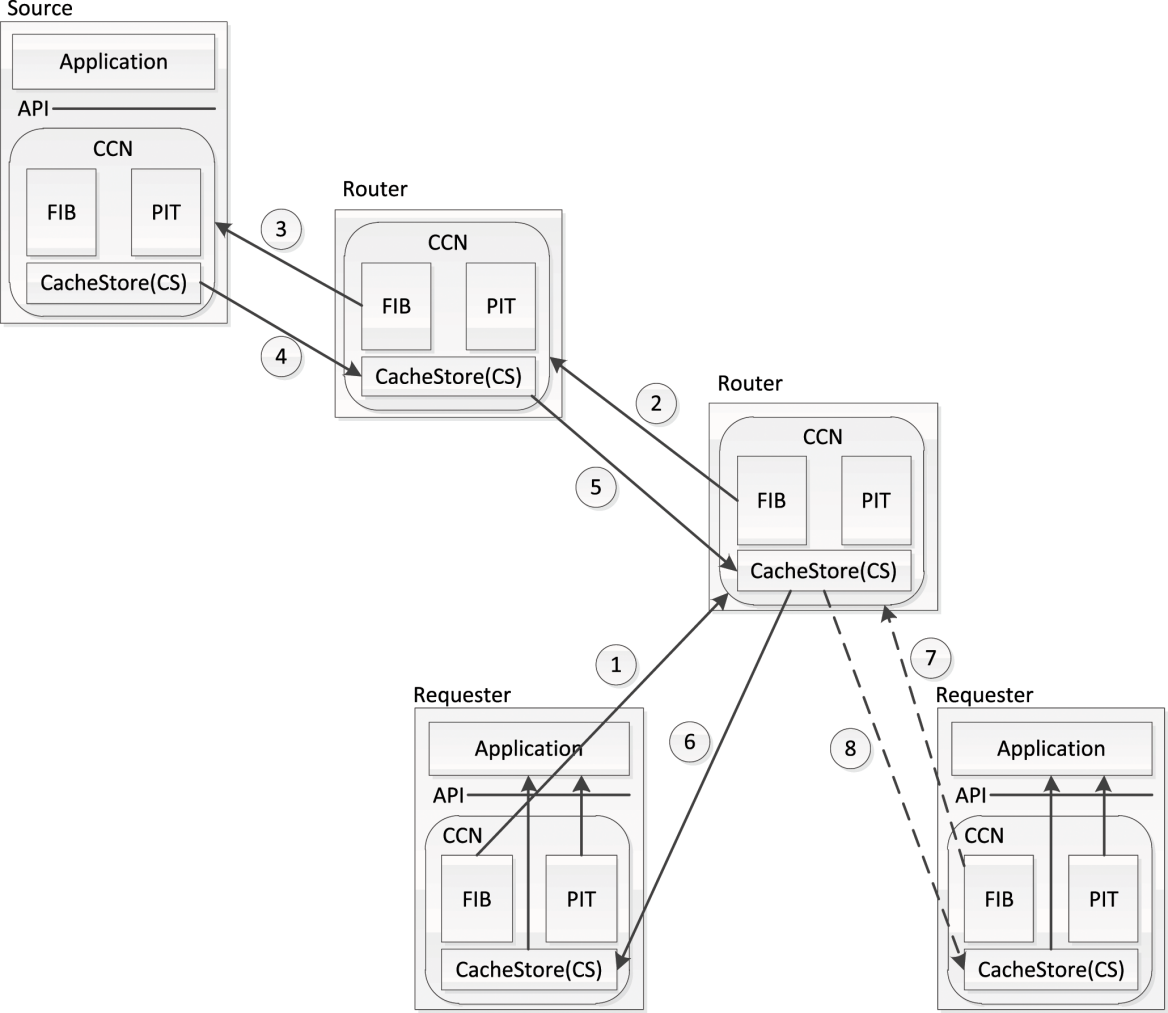
CCN overview.

In CCN, named contents are published at nodes (providers). Each router in CCN contains two main data structures (as shown in Fig 1): FIB (Forwarding Information Base) and PIT (Pending Interest Table). Routing strategies are employed to form the FIB, which directs the corresponding requests (called interests in CCN) for named contents towards the correct providers. Forwarded interests should leave bread crumbs in PIT for sending the returned data down to request generators. Some of the routers with cache function known as cache router in CCN should hold a CacheStore (CS, buffer memory) to store the very objects based on their cache scheme.

When an interest packet arrives on an interface, the cache router first checks its local CS. If the requested data exists in the CS, the router should send it back directly through this interface, and the interest should be discarded, as illustrated by step 7–8. Otherwise, it reviews whether such an interest is already in the PIT, if so, it updates the interface in PIT to assure the correctly reply of chunk. Otherwise, router forwards the request upstream toward content source based on FIB, meanwhile, leaves a bread crumb in PIT (step 1–3). The content source of an object is not only including the content provider, but also the routers caching the object.

The process of relaying replied chunk is relatively simple, since data is only routed through the chain of PIT entries back to the original requester(s) (step 4–6). Whenever a router receives a chunk, it should decide whether to cache or not based on its own caching policy.

The request process is assumed to be the so-called “independent reference model”: the probability of arriving request for a given object only depends on the object’s popularity but not on the sequence of requests that came before. The popularity of content object follows generalized ***Zipf*** law, which have been illustrated in many realistic traffic flows [5]: the request rate *q*(*n*) for the *n*_th_ most popular object is proportional to 1/*n*^*α*^ for some ***Zipf*** parameter *α* (denoted by ***Zipf*(*α*)**). Equivalently, we also assume content requests occur at the instants of independent Poisson processes, and cache router implements LRU replacement policy.

## 3. Cache Scheme Based on Prefetching Operation

A content object can be further divided into many fine-granularity chunks with chunk-level communication model in ICN. An end user should send out the interests for all chunks of a content object to the provider, if he wants to get the whole content object. An interest is corresponding to a chunk, not for a content object.

### 3.1 Problem illustration

As mentioned in section 2, by-path caching is one of the main components in CCN. The interest is forwarded by the routers following the FIB, while leaving a bread crumb in the PIT, which directs the data reply. Once a cache router receives a data, it should decide whether cache or not according to the cache policy.

The stop-and-wait request mode is assumed to be applied in CCN: end user should issue the next chunk immediately only after receiving the reply of the requested chunk. If an end user fetches a content object from the provider passing through three cache routers, and none of the cache routers has cached any chunks of such content object, the hop number for successfully achieving a chunk is accounted for 8. If the average propagation delay of each link for transmitting a chunk is *d*, the propagation delay experienced by the user to get a chunk should be 8*d*. The propagation delay is the main factor of user perceived delay to ignore the processing and transmission time while *d* is large enough. If the chunk number of the content object is *Nc*, the total propagation delay the user experienced will be 8*Nc*d.

For some latency-sensitive flow, such as VoD, it is intolerable for a user to wait for a long time to get the following chunks or the whole object. The bad quality of service will lead to the loss of users, which would decrease profit of network provider.

Obviously, in CCN, all the cache action is activated by the data, which is derived from the user requests in essence. In other words, only after the chunk has been asked by the users not long ago, the chunk should be stored in the cache. Should the follow-up chunks retrieval be fasted to reduce the total latency experience by user? Should the routers prepare for the chunks which will be requested in the near future? The main goal of the cache scheme based on pre-fetch operation proposed is to decrease user perceived latency. The details of the cache scheme based on pre-fetch operation are given in the following.

### 3.2 Pre-fetch operation

A cache scheme should decide what to be cached and the replacement policy. Both fetched and pre-fetched chunks are considered to be cached in cache scheme based pre-fetch operation, and LRU replacement policy is used in our scheme.

The pre-fetch operation as the core function of our cache scheme should answer what to be pre-fetched and how to pre-fetch. We argue that, the probability for the downstream node (or end user) to request the following few chunks in the same content of the requested chunk deems to be rather higher than to request the other chunks far away or from other contents. Then pre-fetching the following chunks of the same content omits complex pre-fetch prediction, which is the main work in web pre-fetch or recommendation system recently. The number of pre-fetch chunks is labelled as pre-fetch window *W*, which is the main factor affecting the latency reduction.

In the following, we focus on how to pre-fetch. As the assumption mentioned in WAVE [17], the original servers as well as cache routers can recommend its downstream cache routers to store the chunks. We also assume the pre-fetch operation can be advised by the upstream nodes. For more efficient prefetching and caching, we need some collaboration among cache routers. In this paper, a cache router suggests the pre-fetching operation to its downstream cache routers by marking the chunk when it forwards the chunk. For this, the pre-fetch flag bit is needed in the chunk (e.g., in the Data packet header in CCN). If a cache router receives a chunk with the non-zero pre-fetch flag, the router is suggested by the upstream node with the pre-fetching operation. A cache router generates the pre-fetch operation only when meets any of the following pre-fetch-driven-modes.

1. cache-driven mode: A downstream request is hit in its local cache
2. data-driven mode: It receives the replied data requested by the downstream nodes (not generated by itself), and the pre-fetch flag in the header is 1.

Note that, the request mentioned in both modes should be received from the neighbour nodes, not issued by its own pre-fetch operation, which avoids the endless self-driving pre-fetch operation.

The cache-driven mode endows each cache router to decide the pre-fetch operation by itself, while the data-driven mode enable the upward nodes to recommend whether the pre-fetch operation should occur at the downstream cache router or not through setting the pre-fetch flag in the chunk packet header, which is the collaborative pre-fetch idea. The pre-fetch flag can be decided by many factors, such as the content popularity, hops to the nearest source (cache or provider), and the priority, etc. However, how to set the pre-fetch flag is beyond the scope of this paper. The pre-fetch flag here is just decided by the hop to the nearest content source.

**Fig 2:**
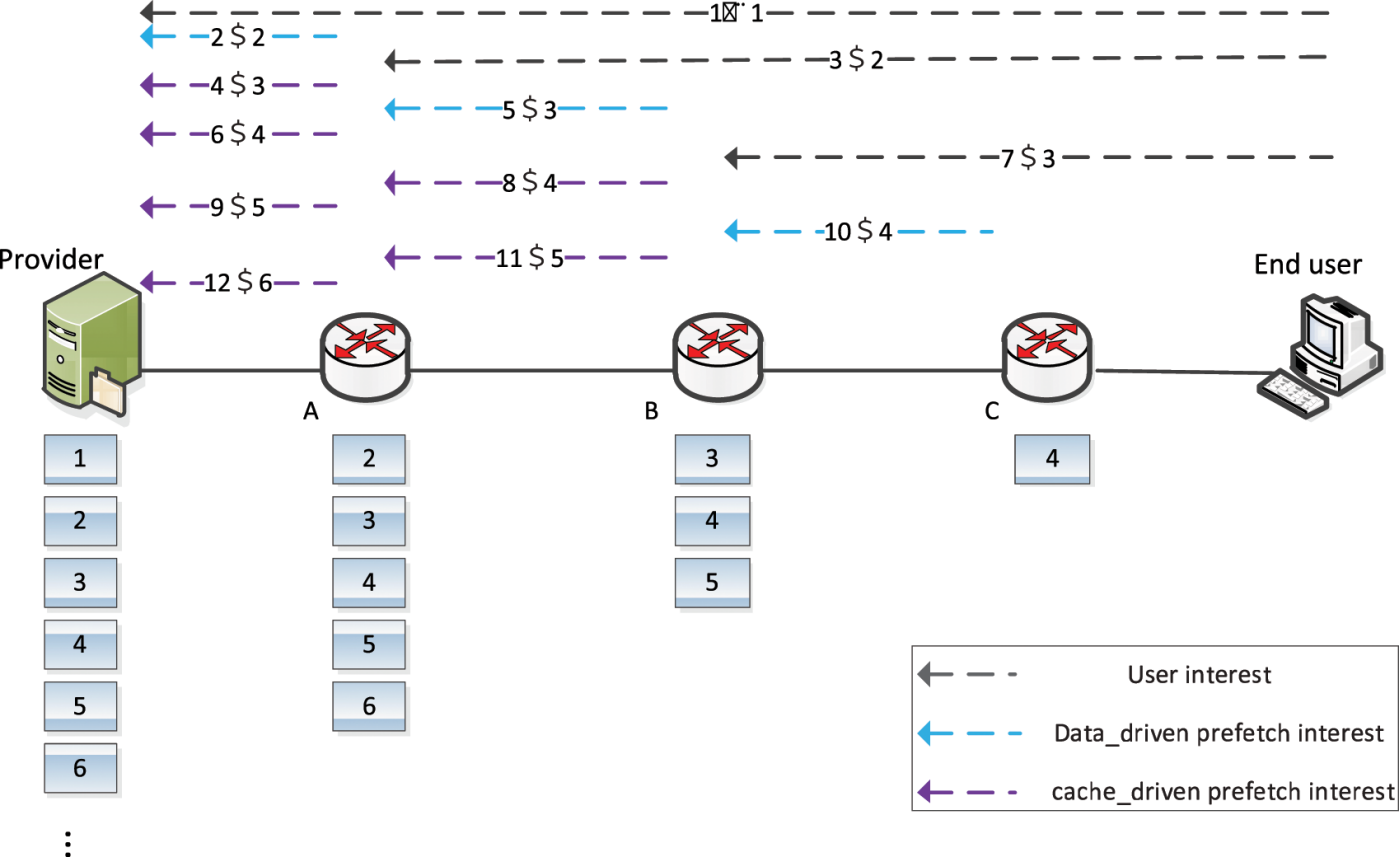
Example of pre-fetch operation.

Fig 2 gives an example of request process for cache scheme based on pre-fetch operation with pre-fetch window *W* = 1 (denoted *W*(1)), while it omits the reply process under the assumption of stop-and-wait request mode. The provider owns the whole chunks of the requested content, depicted by the numeric boxes below. No chunks for the requested content have been cached on the cache routers before. All links have the same communication delay. The pre-fetch flag here is decided only by the hop number to the nearest content source in the header. If the hop equals to 1, the pre-fetch flag is set to be 1. Otherwise, the pre-fetch flag remains 0. The dotted arrows with different colours are used to express the three classes of interests respectively: user interest, data-driven pre-fetch interest, cache-driven pre-fetch interest. The first number on the dotted arrow is the sequence number of event generated; the second one represents the requested chunk number of the content object. The numeric boxes below each cache router are the pre-fetched chunks which should be cached. For simplifying the description, we use interest[i] to represent the request for the *i*_*th*_ chunk, data[i] to denote the reply of interest[i], and (a,b) to show the event on dotted arrow.

The request process of cache scheme based on pre-fetch operation is given as below.

Step 1: The end user firstly sends out the user request interest[1], which should be forwarded towards the provider based on the CCN communication process (as shown in (1, 1))

Step 2: After received the data[1] (which is omitted in Fig 2 and should occur between (1,1) and (2,2)), router A should cache and forward data[1] downwards according to standard CCN replying process. Meanwhile, because the pre-fetch flag in the header is 1 (hop is 1), router A generates and sends out interest[2] to the provider for pre-fetching the *2*_*nd*_ chunk based on data-driven mode, as shown in (2,2). Because of the LRU cache replacement, router A should cache the data[2] after received it, which is the first chunk in numeric boxes below router A. But data[2] should not be forwarded downstream without arriving interest.

Step 3: Interest[2] originated from end user (3,2) should be hit in local CS of router A because of its previous pre-fetch operation, which enables router A to pre-fetch the next chunk according to cache-driven mode, the interest[3] of event (4,3). Data[2] will be forwarded back to requestor by router A.

Step 4: The reply of data[2] allows router B to generate data-driven pre-fetch interest[3] (see event(5,3)), since the hop between router B to the nearest source of data[2] (router A) is 1, that is, the pre-fetch flag is 1. Then such interest[3] should be hit on router A for step 3. Router A should issue cache-driven pre-fetch interest[4] upstream to provider (6,4).

Step 5: User request interest[3] (7,3) can be hit on router B for event (5,3), which drives the cache-driven pre-fetch interest[4] of router B (see (8,4)). Finally in turn to cache-driven pre-fetch interest[5] of router A (9,5).

Step 6: Similarly, the reply of data[3] enable router C to pre-fetch data[4] (10,4). Cache-driven pre-fetch interest[5] (11,5) and [6] (12,6) are sequentially activated on router B and A.

After step 6, user could gain the following chunks directly on router C. At the same time, the following user interest[i] could trigger a cache-driven pre-fetch interest[i+1], [i+2] and [i+3] on router C, B and A, respectively.

Comparing to the previous average hop *8* and the total hop *8Nc*, the hops in cache scheme based on pre-fetch operation are shrinking to be (12+2 *Nc*)/ *Nc* and (12+2 *Nc*). When *Nc* is much larger than 3, the average hop reduced is 6.

The above pre-fetch process is also applicable for other pre-fetch window *W*. Then the number of pre-fetch router sent out is set to be *W*, when router performs pre-fetch operation.

### 3.3 Qualitative analysis

In this section, we first give the mathematical model for network delay comparison between CCN architectures with pre-fetching and without pre-fetching. And the result of average latency comparison is given to show the validity of the pre-fetching based caching scheme in CCN.

Let *N* be the number of total content objects. Each object is composed by *K* chunks. The request rate of contents follows *Zipf* distribution. Therefore, the requested probability for *n*_*th*_ most popular content *p*(*n*) is given as *p*(*n*) = *A* / *n*^*α*^, where $A=\sum_{n\in [1,N]}1/n^{\alpha }$. *α* is the *Zipf* parameter. It is reasonable to apply the independent reference model at each router, because its request process results from the superposition of many independent overflow processes, each of that contributes a small fraction of overall request. In this configuration, the occupancy states of cache routers can reasonably be assumed to be statistically independent.

The total request rate on a router is the sum of rates requested by neighbouring nodes and generated by itself. If the total request content rate on a node is *q*, the request rate for content *n* should be *q*(*n*) = *q*×*p*(*n*). The request rate for each chunk in content *n* is also *q(n)*, for the chunks of the same content object with the same request rates. Given the delivery of content from server to user should traverse routers *R*_*M*_, *R*_*M-1*_, …, *R*_*1*_; and the delay for each hop is *T*_*hop*_.

We firstly give the quality analysis on standard CCN architecture without pre-fetching. Because all of the chunks belonging to the same content are assumed to have the same request rate, the hit ratios for all chunks in a router *i* are the same. For chunk *k* of content *n*, the hit ratio on router *i* is *P*_*i*_(*n*, *k*). For any chunks *m* of content *n*, the hit ratio satisfied that *P*_*i*_(*n*, *k*) = *P*_*i*_(*n*, *m*) = *P*_*i*_(*n*). On the path from server to a user with *M* routers, all requested chunks can be attained from server, that is, *P*_*M*+1_(*n*) = 1.

With LRU, we use an approximation proposed by Che et al. [5] to estimate the cache hit ratio. If the cache capacity is *C*, the hit ratio *P*_*i*_(*n*, *k*) for chunk *k* of content *n* with request rate *q(n)* can be written as $P_{i}(n,k)=1-e^{-T_{C}\times q(n)}$, for some parameter *T*_*C*_. The capacity should hold all cached object, that is, $C=\sum_{n\in [1,N]}K(1-e^{-T_{C}\times q(n)})$.

To model the delay for the chunk *k* of content *n* in CCN, it is known that only the missing requests should be forwarded upward. That is, the request *k* reaches on a router *i* only when the interest is missed in all of the downstream routers. Let *AP*_*i*_(*n*, *k*) to be the arrival probability of request *k* reaches on a router *i*, which can be expressed as

$$AP_{i}(n,k)=\prod_{j<i}\left(1-p_{j}(n,k)\right)$$ (1)

*T*_*CCN*_(*n*, *k*) is the delay for retrieval of the chunk *k* in content *n*, which can be described as the sum of all situations that the interest *k* is hit on each router. Because of the all the chunks in the same content get the same hit ratio on a router. So the average delay for the chunks in content *nT*_*CCN*_(*n*) is also *T*_*CCN*_(*n*, *k*). That is,

$$T_{CCN}(n)=T_{CCN}(n,k)=\sum_{i\in [1,M+1]}2iT_{hop}\times p_{i}(n,k)\times AP_{i}(n,k)$$ (2)

The term 2*iT*_*hop*_ is the round-trip-time between router *i* and the user. The average delay for user to get any chunk of any content should be expressed as

$$T_{CCN}\_avg=\sum_{n\in [1,N]}p(n)\times T_{CCN}(n)$$ (3)

Then the network latency for cache scheme based on pre-fetching is derived. Because of the pre-fetching operation, the request rate on each router is different from the one without pre-fetching, which further causes the change of the hit ratio on routers, and finally leads to the delay variation.

*W*(1) as an example is analysed here to show the effect of the cache scheme based on pre-fetch. The other pre-fetch windows can be deduced in the same way. The online cache is functionally supposed to be composed by common cache and pre-fetching cache under the pre-fetching operation. The pre-fetched without requested downstream chunks should be stored in pre-fetching cache. The pre-fetch operation is enabled after the hit rate of common cache has been stabilized. The chunks in pre-fetching cache should be hold for a time to ensure the arrival of request downstream. The chunk should be shifted to common cache, if the chunk is requested by downstream node. The overdue of pre-fetched chunk means that the chunk should not be requested by downstream node obviously, so the chunk should be deleted from pre-fetching cache when the stored time exceeds for a limit. Although the functions of common cache and pre-fetching cache are different, they share the same buffer memory with almost the same eviction time in LRU.

For chunk *k* of content *n*, the hit rate on router *R*_*i*_’s common cache is $P_{i}^{com}(n,k)$. It is reasonable assumed that the hit rates for a given chunk on all routers’ common cache are almost the same. That is, $P_{i}^{com}(n,k)=P_{j}^{com}(n,k)$ (∀*i* ≠ *j*), for router *R*_*i*_ is different from *R*_*j*_. With LRU approximation mentioned before, the hit ratio of common cache can be estimated. If the common cache capacity is *C*, the hit rate $P_{i}^{com}(n,k)$ for chunk *k* of content *n* with popularity *q(n)* can be written as $P_{i}^{com}(n,k)=1-e^{-T_{C}\times q(n)}$, for some parameter *T*_*C*_. The capacity should hold all cached objects, that is, $C=\sum_{n\in [1,N]}K(1-e^{-T_{C}\times q(n)})$. The hit rate of all chunks on server is 1, that is, $P_{M+1}^{com}(n,k)=1$. Let $P_{i}^{pre}(n,k)$ to be the possibility for router *R*_*i*_ to pre-fetch chunk *k* of content *n*, which can also be seemed as the pre-fetch hit rate. If chunk *k* has been pre-fetched by router *R*_*i*_ before the downstream requesting, the interest should be hit. The pre-fetch operation should not occurred on server, therefore, $P_{M+1}^{pre}(n,k)=0$.

If the total hit rate on router *R*_*i*_ is *H*_*i*_(*n*, *k*) (*i* ∈ [1, *M* + 1]), that is,

$$H_{i}(n,k)=P_{i}^{com}(n,k)+P_{i}^{pre}(n,k)$$ (4)

The hit rate of pre-fetch cache $P_{i}^{pre}(n,k)$ is calculated in the following section. Router *R*_*i*_ (*i* ∈ [1, *M*]) pre-fetches chunk *k* only when the following prerequisites are met: a) At first, chunk *k* has not been stored in common cache. The possibility is calculated as $\left(1-P_{i}^{com}(n,k)\right)$; b) secondly, one of the driven modes is satisfied. The downstream request for chunk *k*-1 must arrive and hit at router *R*_*i*_ in cache-driven mode; while in data-driven mode, the downstream request for chunk *k*-1 must arrive and be hit at router *R*_*i*_+1, when the pre-fetch flag is determined by the hop in the header. For router *R*_*i*_, the downstream request is the pre-fetch request generated by downstream routers, or the user request. Hence,

$$\begin{array}{l} P_{i}^{pre}(n,k)=(1-P_{i}^{com}(n,k))[(\sum_{0<j<i}P_{j}^{pre}(n,k-1)hit_{j,i}(n,k-1)+P_{i}(n,k-1))\\ \;+(\sum_{0<j<i+1}P_{j}^{pre}(n,k-1)hit_{j,i+1}(n,k-1)+P_{i+1}(n,k-1))] \end{array}$$ (5)

In expression (5), $\sum_{0<j<i}P_{j}^{pre}(n,k-1)hit_{j,i}(n,k-1)+P_{i}(n,k-1)$ and $\sum_{0<j<i+1}P_{j}^{pre}(n,k-1)hit_{j,i+1}(n,k-1)+P_{i+1}(n,k-1)$ are the probabilities for downstream request *k*-1 arriving and being hit at router *R*_*i*_ and *R*_*i*_+1, respectively. If a pre-fetch request *k* is generated by router *R*_*i*_, the conditional probability for the request *k* arrives and hits on upstream router *R*_*j*_ is *hit*_*i*,*j*_(*n*, *k*). Where,

$$hit_{i,j}(n,k)=H_{i}(n,k)\prod_{i<t<j}(1-H_{j}(n,k))$$ (6)

The delay expectation *T(n*,*k)* for user to get the chunk *k* of content *n* should be

$$T(n,k)=\sum_{i\in [1,M+1]}T_{i}(n,k)$$ (7)

Where, *T*_*i*_(*n*, *k*) is the delay expected for user to get chunk *k* from router *i*. Let *P*_*i*_(*n*, *k*) and *D*_*i*_(*n*, *k*) to be the probability and delay experienced respectively, when user sends out the request *k* be hit at router *R*_*i*_. Then

$$\begin{array}{l} T_{i}(n,k)=\prod_{j<i}\left(1-H_{j}(n,k)\right)\times [P_{i}^{com}(n,k)\times D_{i}^{com}(n,k)+P_{i}^{pre}(n,k)\times D_{i}^{pre}(n,k)]\\ \;=\prod_{j<i}\left(1-H_{j}(n,k)\right)\times [P_{i}^{com}(n,k)+P_{i}^{pre}(n,k)]\times [\frac{P_{i}^{com}(n,k)}{P_{i}^{com}(n,k)+P_{i}^{pre}(n,k)}\times D_{i}^{com}(n,k)+\frac{P_{i}^{pre}(n,k)}{P_{i}^{com}(n,k)+P_{i}^{pre}(n,k)}\times D_{i}^{pre}(n,k)]\\ \;=P_{i}(n,k)\times D_{i}(n,k) \end{array}$$ (8)

If the content *k* request by user is hit on the common cache of *R*_*i*_, the delay is represented by $D_{i}^{com}(n,k)$; otherwise, if the request is hit at the pre-fetch cache of *R*_*i*_, the delay is $D_{i}^{pre}(n,k)$. Then

$$P_{i}(n,k)=H_{i}(n,k)\prod_{j<i}\left(1-H_{j}(n,k)\right)$$ (9)

$$D_{i}(n,k)=\frac{P_{i}^{com}(n,k)}{H_{i}(n,k)}\times D_{i}^{com}(n,k)+\frac{P_{i}^{pre}(n,k)}{H_{i}(n,k)}\times D_{i}^{pre}(n,k)$$ (10)

We calculate the delay expectation for user to get the chunk *k* from router *R*_*i*_ in the following.

It is more complex for solving *D*_*i*_(*n*, *k*). Because when user request *k* arrives at router *R*_*i*_, it can be hit on the common cache. Similarly, it can be stored on the pre-fetch cache; or it has been pre-fetched but has not got the replied chunk by *R*_*i*_. If the pre-fetch request generated by *R*_*i*_ is hit by a upstream router *R*_*t*_, it also can be hit by common cache or pre-fetch operation of *R*_*t*_, which is the repeat of the previous procedure. Obviously, it is too difficult to get the accurate value of *D*_*i*_(*n*, *k*). However, we can always find the nearest source node *S* (*i* ≤ *S* ≤ *M* + 1) of chunk *k* from *R*_*i*_. The source node is the node storing the chunk *k* in its common caches. Therefore, we turn to get the worst-case delay. That is, if the user request is hit by pre-fetch operation of *R*_*i*_, the routers between *R*_*i*_ and *S* are assumed without pre-fetch operation. Under this situation, let *Wait*_*i*,*S*_(*n*, *k*) be the waiting time after request arriving at *R*_*i*_.

If *S* is *R*_*i*_, that is, user request is hit at the common cache of router *R*_*i*_, *Wait*_*i*,*S*_(*n*, *k*) = 0. If *S*>*i*, *R*_*i*_ is the nearest pre-fetching node from user. The generation time of pre-fetch request *k* almost is the same as the replied time of chunk *k-1* on *R*_*i*_. Hence, *Wait*_*i*,*S*_(*n*, *k*) = max{0, (*S* − *i*) − (*i* − 1)} = max{0, *S* − 2*i* + 1}. Combining the above two situations, the waiting time on *R*_*i*_ can be expressed as

$$Wait_{i,S}(n,k)=\text{max}\{0,(S-i)-(i-1)\}=\text{max}\{0,S-2i+1\}$$ (11)

The probability for the upstream router *R*_*j*_ of *R*_*i*_ acting as *S* is *PS*_*i*,*j*_(*n*, *k*), then

$$PS_{i,j}(k)=\{\begin{array}{l} P_{i}(n,k)/H_{i}(n,k),\;if\;i=j\\ P_{i}^{pre}(n,k)/H_{i}(n,k)\times hit_{i,j}^{com}(n,k),\;else \end{array}$$ (12)

Therefore,

$$D_{i}(n,k)=\sum_{j\in [i,M+1]}PS_{i,j}(k)\times 2T_{hop}\times [(i+1)+wait_{i,j}(n,k)]$$ (13)

Substituting the expression (9)-(13) into (8), the *T*_*i*_*(n*,*k)* should be

$$\begin{array}{l} T_{i}(n,k)=\prod_{j<i}\left(1-H_{j}(n,k)\right)\times [P_{i}^{com}(n,k)+P_{i}^{pre}(n,k)]\\ \;\times [\sum_{j}PS_{i,j}(n,k)\times 2T_{hop}\times [(i+1)+wait_{i,j}(n,k)]] \end{array}$$ (14)

Then, the delay *T(n*,*k)* for user getting chunk *k* of content *n* should be attained after substituting (14) into (7).

The average delay for getting any chunk of content *n* is

$$T\_avg(n)=\sum_{k\in [1,K]}T(n,k)/K$$ (15)

Thus, the average delay for user to get any chunk of any content should be

$$Delay\_avg=\sum_{n\in [1,N]}p(n)\times T\_avg(n)$$ (16)

To show the validity of our method, the parameters are given in the following. The content number *N* is 10000, and the chunk number *K* is 10. The number of cache routers *M* is 3. The cache capacities deployed on cache routers are all set from 100 to 1000. And the request rates originated from users on all routers are set as 50. The *Zipf* parameter is 0.8. Table 1 gives the results compared between the standard CCN with LRU and the cache scheme based on pre-fetch. *T*_*hop*_ is 10 ms. Table 1 gives the results compared between the standard CCN with LRU and the cache scheme based on pre-fetch. Obviously, under different cache deployment, the average delay under pre-fetching *Delay_avg* is always smaller than *T*_*CCN*_*_avg* without pre-fetching.

**Table 1:** 

Average latency comparison	Cache capacity		
	100	500	1000
*T*_*CCN*_*_avg*(ms)	72	58	47
*Delay_avg*(ms)	48	33	29

The other results compared with the delay without pre-fetch operation will be given in the following section through the simulation.

## 4. Performance Evaluations

The performance is shown in both the results of analysis and simulation. According to the former qualitative analysis, the efficiency of cache scheme based on pre-fetch operation can be demonstrated. While the performance evaluation is further conducted by simulation in three aspects: i) the average latency; ii) the average hops; iii) the hit rate. The simulation results will be influenced by cache capacity, ***Zipf*** distribution parameters *α* and pre-fetch window size *W*.

### 4.1 Simulation scenarios

**Fig 3:**
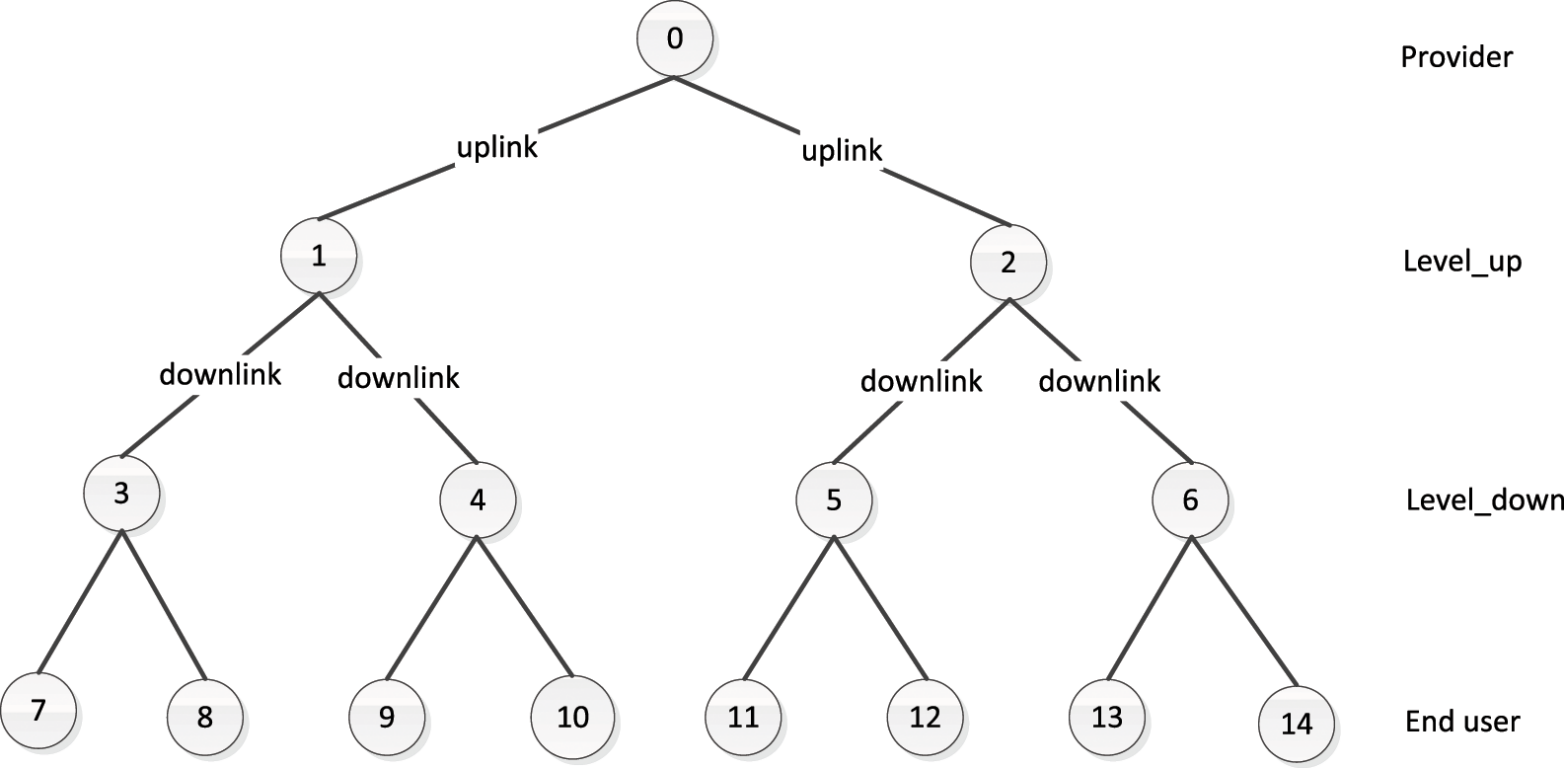
Simulation topology.

Binary trees with 15 nodes are deployed, as shown in Fig 3. The leaves behave as clients which connected by end users who should generate the requests. The root node is the only content provider. The other intermediate nodes are cache routers with the same cache capacity, which can trigger the pre-fetch operations. The cache routers are named as level_down and level_up routers, respectively, according to the location in the topology. The uplinks refer to the links between root to level_up cache routers, while the links between level_up and level_down cache routers are named as downlink. All the links are set to be with infinite bandwidth and the same propagation delay, which set to be 100ms. As a rule of thumb, the shortest path routing is used in the experiment.

We study the cache scheme based on pre-fetch operation under an open source software ccnSim [30], chunk-level simulator of CCN. We assume end user should sequentially request for whole chunks of a content object. Stop-and-wait (no-overlap) request manner is employed. The content number and the average chunk number per content are set to be 10^6^ and 10, respectively, which are in accordance with Youtube-like internet catalogues [27]. And all the chunks have the same size. The unit of cache capacity is chunk. The requests of the content-level are generated followed by a Poisson process. The request rate (lamda) of each client is 500/s. Two main factors, i.e., cache size and ***Zipf*** parameter *α*, are adjustable in the experiments. At first, cache size is varied uniformly from 100 to 10^6^ chunks for all cache routers. Secondly, we vary the Zipf parameter with 0.8, 1.2 and 1.6. The pre-fetch window is set to be 1, and then varies from 2 to 8 with an increment of 2.

We repeat simulations over 20 times for each combination of cache size and ***Zipf*** parameter for each pre-fetch window. Each simulation is ended by either the convergence of hit rate for all cache routers, or simulation time is over 2 hours after cache stuffed.

We should consider three performance metrics:

1. Hit rate. Hit rate is often used to measure the caching performance. In our experiments, we study the hit rates of different cache level influenced by pre-fetching operation.
2. Average latency perceived by users. Our main goal of cache scheme based on pre-fetch operation is to reducing user-perceived delay. The average latency is composed by the transmission delay, propagation delay and processing delay. Because the transmission delay and processing delay are too trivial to be ignored comparing to propagation delay in our simulation, here we only care about the propagation delay.
3. Average hops. As we demonstrated in section 3, the pre-fetch flag is decided by the hops carried by the header. So the average hop is another important metric for cache scheme based on pre-fetch operation.

### 4.2. Performance results

**Fig 4:**
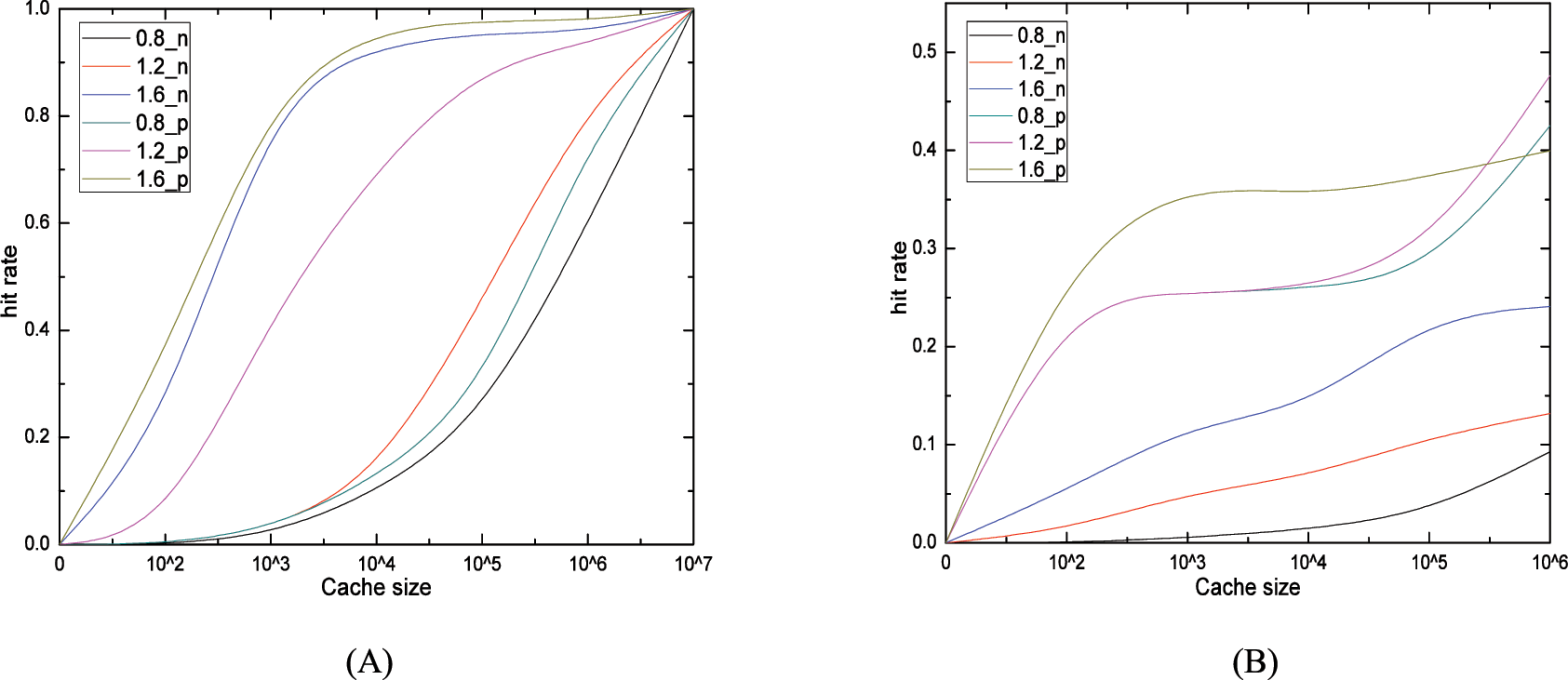
**Comparison of hit rate between un-prefetch and W(1) (a) hit rate of level_down. (b) hit rate of level_up**.

To illustrate the effectiveness of cache scheme based on pre-fetch operation, we firstly compare the results without and with pre-fetch operations (pre-fetch windows is 1, W(1)). Fig 4 gives the hit rate comparison with different ***Zipf*** parameters and different cache sizes, deduced by qualitative analysis. The hit rate of level_down and level_up are presented in Fig 4(A) and 4(B), respectively. The “num” in line mark “num_n/p” represents the ***Zipf*** parameters, while the “n/p” represent without or with pre-fetch operation. The hit rates show three main trends: ① the hit rate with pre-fetching is significant higher in both cache levels under different ***Zipf*** parameters; ② no matter with pre-fetch operation or not, the hit rate rises with the incremental of ***Zipf*** parameter and ③ cache size in both level_up and level_down. When ***Zipf*** parameter is 1.2, the gap between pre-fetch and noprefetch is larger than the other situations in Fig 4(A). When the cache capacity is smaller than 5*10^3^, the level_up hit rate for ***Zipf***(1.2) and ***Zipf***(0.8) is almost overlapping in Fig 4(B). Meanwhile, when cache size is closer to the 0.1 of total chunk number, both level_up hit rate for ***Zipf***(1.2) and ***Zipf***(0.8) are slightly higher than ***Zipf***(1.6).

**Fig 5:**
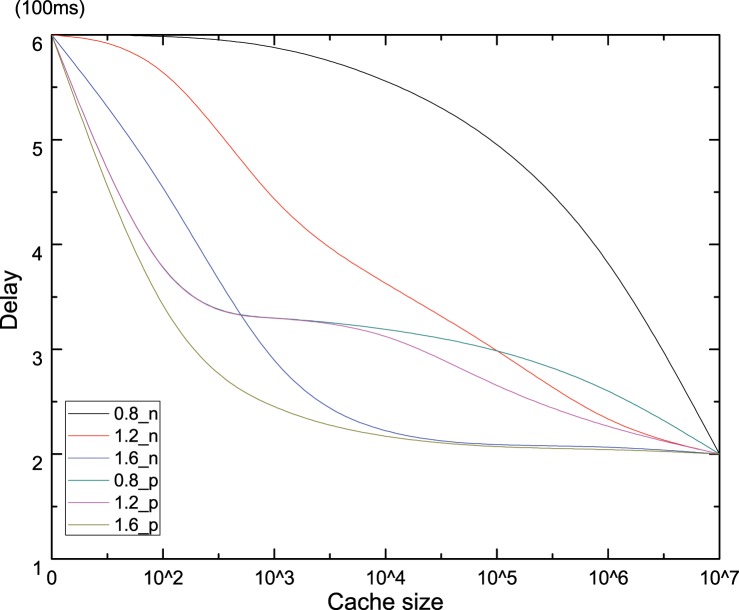
Average delay perceived by end user.

**Fig 6:**
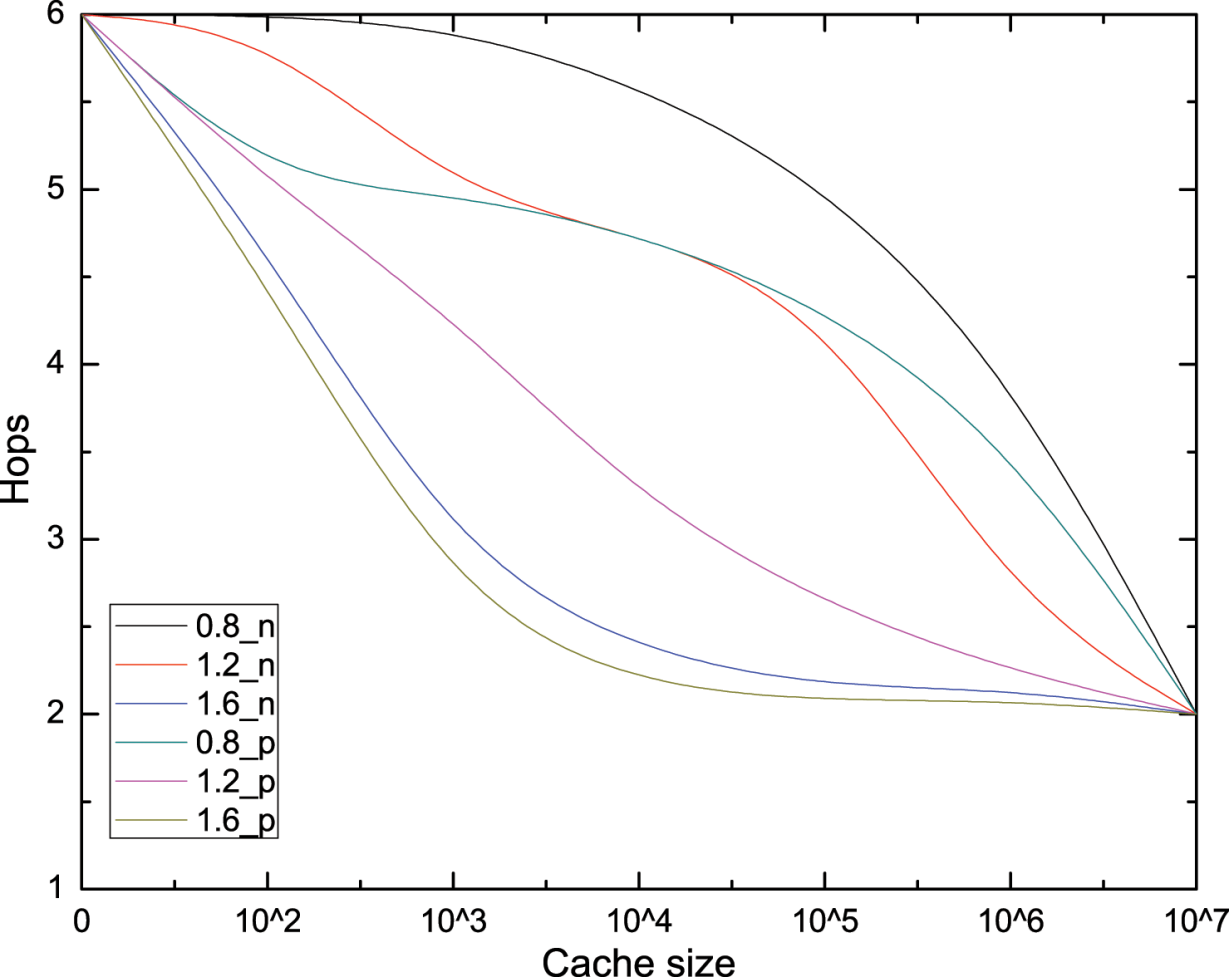
Average hops.

Figs 5 and 6 show the results of cache based on pre-fetching operation in terms of user perceived average delay and hops under increasing of cache capacity and ***Zipf*** parameter. Firstly, it is can be concluded that both the average delay and hops with pre-fetch operation are always lower than that without pre-fetch operation, which demonstrates that pre-fetch operation can effectively decrease the average delay and hops and conforms to our mathematical analysis in section 3.3. After normalized the propagation delay of each link, the average delay shows the same declining trend with hops. However, the average delay with pre-fetch operation is always smaller than average hops, as shown in Figs 5 and 6. Because when a cache router received pre-fetched data after downstream interest for a chunk, the hops in header should not to be reset, while the delay is significantly reduced. The max differences between pre-fetch and no-prefetch are about 300 ms, 250 ms, 100 ms, with the cache capacity is near 5000,1000,500 for ***Zipf***(0.8), ***Zipf***(1.2) and ***Zipf***(1.6) respectively, which keeps accordance with reality that caching capacity is usually much smaller than the overall chunk population. So the pre-fetch operation is an effective method to improve the performance for end user.

**Fig 7:**
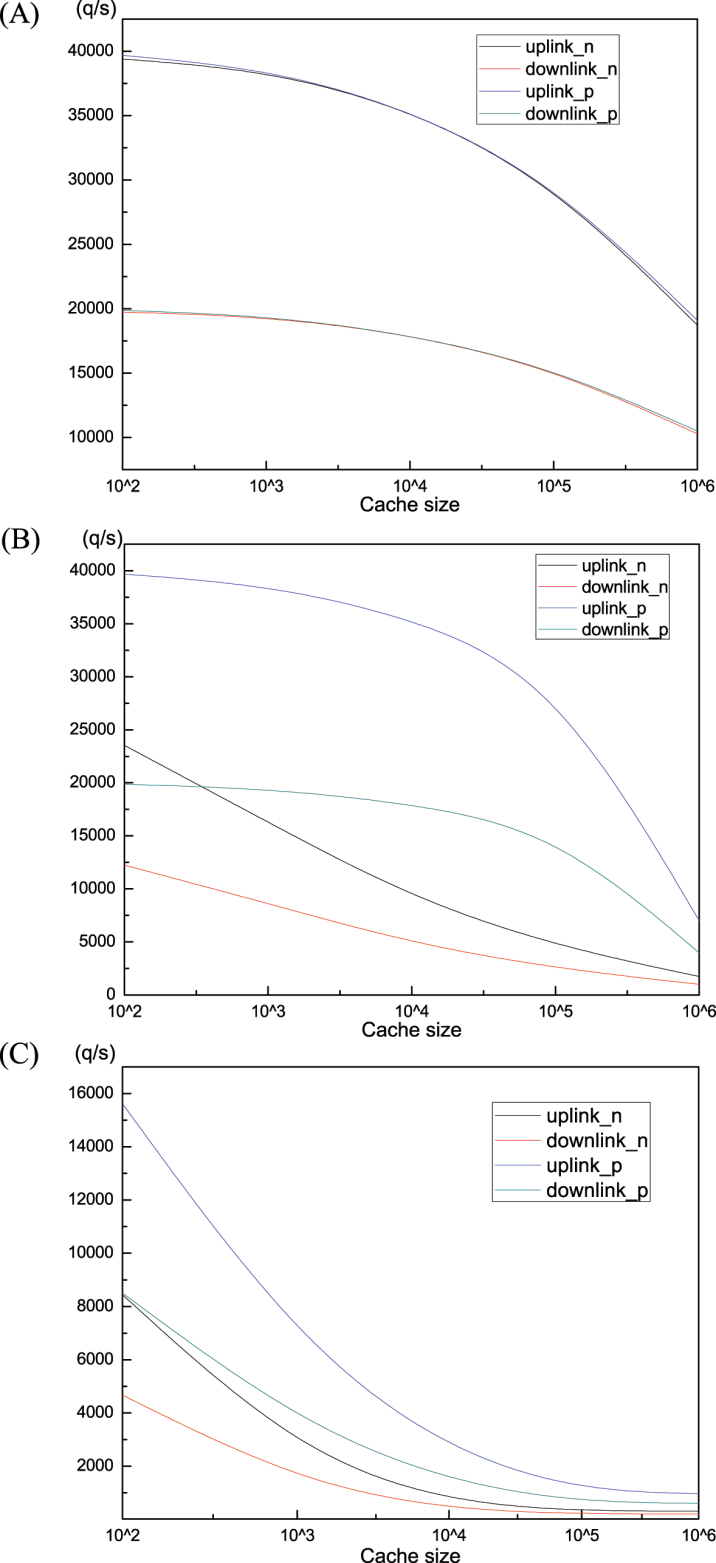
**Bandwidth (a) *Zipf*(0.8). (b) *Zipf*(1.2). (c) *Zipf*(1.6)**.

We plot the corresponding bandwidth requirement versus cache capacity for different ***Zipf*** parameters in Fig 7. The”uplink/downlink_n/p” means the uplink or downlink bandwidth requirement without or with pre-fetching. It can be seen that, with cache size increasing, the bandwidth needed is reduced for all ***Zipf*** parameters. The pre-fetch operation shouldn’t raise the bandwidth requirement for ***Zipf***(0.8) as described in Fig 7(A). However, the bandwidths needed of uplink and downlink under pre-fetching are both much larger for ***Zipf***(1.2) and ***Zipf***(1.6), as shown in Fig 7(B) and 7(C). When ***Zipf*** parameter is 1.2, the bandwidth required of pre-fetch operation is always over twice more than without pre-fetching. Especially, when cache size is about 10^4^, the bandwidth demanded of pre-fetching is almost four times larger than without pre-fetching. However, the gap between pre-fetching and no-prefetching is narrowing with the incremental of cache capacity with ***Zipf***(1.6).

**Fig 8:**
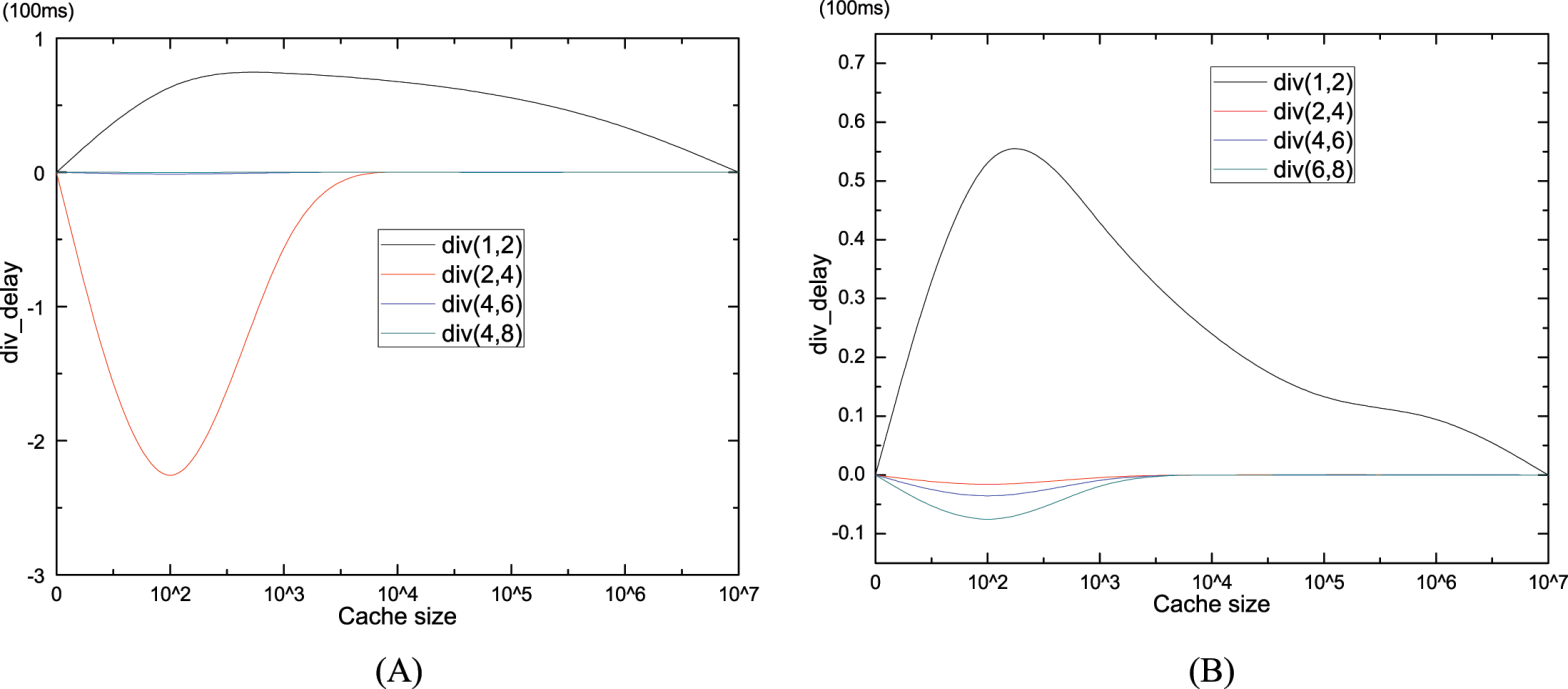
The delay diversity for different pre-fetch windows. (a) delay diversity for *Zipf*(0.8). (b) delay diversity for *Zipf*(1.2).

Finally, we consider average delay for different *W*. Because the distinctions among delays for some *W* are too trivial to be noticed, we adopt the diversity of average delay to show the influence of W, as shown in Fig 8. Div(n1, n2) is the average delay for W(n1) minus that of W(n2), where n1 and n2 are the pre-fetch window size. The average delay for W(2) is always lower than W(1) for both ***Zipf***(0.8) and ***Zipf***(1.2). Meanwhile, when the cache size smaller than 5000, the delay for W(2) is also smaller than any other pre-fetch windows. The maximum difference is about 2 in ***Zipf***(0.8) (in Fig 8A)), but 0.1 in ***Zipf***(1.2) (in Fig 8B)).W(2) is the optimal pre-fetch window for minimal average delay, which conforms with the conclusion of [27] that enlarging pre-fetch window may worse the performance (average delay).

## 5. Conclusion

The cache scheme based on pre-fetch operation of ICN is proposed in this paper. The following chunks of the request content object should be pre-fetched to short the latency user experienced. Two pre-fetch driven modes, cache-driven and data-driven modes, are suggested. When meets one of the driven modes, router should perform pre-fetch operation. Mathematical model is formulated to qualitatively analyse the network latency for both standard CCN without and with pre-fetching operation. The calculation results demonstrate that the pre-fetch operation can reduce the average latency passed for any chunk. In the performance evaluation, simulation results illustrate cache scheme based on pre-fetch operation should always be efficient in reducing the user perceived latency and average hops. The ***Zipf*** parameter and cache capacity have impacts on the performance of cache scheme based on pre-fetch operation. Meanwhile, the pre-fetch window size will also affect the network latency. Our near future work will research the dynamically adjustment of pre-fetch windows for better performance, and collaborative pre-fetch scheme based on pre-fetch flag decision.
